# A Low Mean Closing Load and a Decrease in Load Change at the Tip Increase the Comfort of Scissors

**DOI:** 10.7759/cureus.51900

**Published:** 2024-01-08

**Authors:** Gaku Ota, Yuji Kaneda, Yoshitaka Maeda, Kosuke Oiwa, Ryusuke Ae, Mikio Shiozawa, Hisanaga Horie, Naohiro Sata, Hiroshi Kawahira

**Affiliations:** 1 Department of Surgery, Jichi Medical University, Division of Gastroenterological, General and Transplant Surgery, Shimotsuke, JPN; 2 Medical Simulation Center, Jichi Medical University, Shimotsuke, JPN; 3 Department of Information and Management Systems Engineering, Nagaoka University of Technology, Nagaoka, JPN; 4 Center for Community Medicine, Jichi Medical University, Division of Public Health, Shimotsuke, JPN; 5 Department of Surgery, Tochigi Medical Center Shimotsuga, Tochigi, JPN

**Keywords:** kansei engineering, tacit knowledge, quality control, surgical instruments, cooper scissors

## Abstract

Introduction

During surgery, surgeons intuitively recognize when they are using dull scissors and find them difficult to use. The purpose of this study was to objectively evaluate the physical characteristics of scissors and the comfort reported by surgeons to develop objective quality control standards for scissors used in surgery.

Methods

Sensory and measurement tests were conducted to evaluate the comfort and physical characteristics of ten pairs of Cooper scissors. As a sensory test, thirty-one volunteer surgeons opened and closed the scissors and selected three that felt comfortable and three that were uncomfortable. The results were scored. For measurement, a load was applied to the handle of the scissors. The load pressure and displacement of the width between each handle when the scissors were closed were measured.

Results

A strong negative correlation was found between the total comfort score and the mean load value between sensory and measurement tests (r=-0.717, p=0.0195). The correlation between the total score and the change in load at the tip showed a moderate negative correlation (r=-0.687, p=0.0282). Multiple regression analysis showed that the change in load at the tip was an independent factor affecting the total score.

Conclusions

Surgeons consider scissors with a low mean load required to close the scissors and a small change in load at the tip to be comfortable. The mean load on scissors and the change in load at the tip should be considered in the development of quality control standards for scissors used in surgery.

## Introduction

The quality of surgical instruments is essential because it affects surgical outcomes, but many surgeons have encountered scissors that do not cut well [1]. Surgeons expect that the sensation when using scissors will be similar among different pairs of scissors used during an operation, which supports the need for objective measurements of scissors function.

Scissors have existed for many years and have been empirically improved to their current shape [2]. Among medical devices, surgical instruments made of steel, including scissors, are considered to be of low risk to the human body but are controlled according to standards set by regulatory agencies (for example, the Pharmaceuticals and Medical Devices Agency and the US Food and Drug Administration). Despite being important surgical instruments, local structural differences exist in small steel items, even if they are made by the same manufacturer and of the same shape [1]. It is necessary to clarify the evaluation standards for producing scissors used in surgery to act in a uniform manner.

Manufacturers inspect their products according to their own standards before shipment. All products are checked for appearance and function. Appearance inspections are conducted visually or using magnifiers and instruments used to check dimensions, surface treatment, indications such as maker's markings, and others. Functional inspections are conducted when cutting using specified materials. Differences exist even if the manufacturing lots are the same. It is possible that there are differences between the standards sought by surgeons during use and those set by manufacturers during manufacture. The surgeon's standard and the industrial standard should be matched by conducting and comparing objective results of comfort and measurement tests on the same lot of the same product.

Research on scissors used in surgery has undergone a resurgence due to the need for virtual reality (VR) systems and surgical robot development [3-7]. Ergonomics has also been considered for scissors in recent years. Being comfortable for the surgeon who actually uses them is important to reduce mental strain and maximize surgical efficiency and safety [8-10]. There is a paucity of research on the relationship between surgeons' comfort when using scissors and their physical characteristics.

The goal of the present study is to clarify objective factors that affect the performance of scissors used during surgery. Sensations transmitted to the hand when cutting with scissors are a combination of two major factors: the force produced by shearing the object to be cut and the force produced by the friction between the two blades of the scissors [3]. The sensation due to shearing the object to be cut depends on the nature of the object. The force generated by the overlapping blades is an element of the individual scissors themselves. In this pilot study, to objectively investigate the characteristics of scissors themselves, the same lot of scissors from the same manufacturer was examined regarding their physical characteristics and the comfort the surgeon feels when the scissors are only opened and closed without actually cutting anything.

## Materials and methods

Sensory and measurement tests were conducted to evaluate the comfort and physical characteristics of scissors. This study was approved by the Institutional Review Board of Jichi Medical University Hospital (approval number: 21-78), and written informed consent was obtained from each participant before starting the study.

Scissors

**Figure 1:**
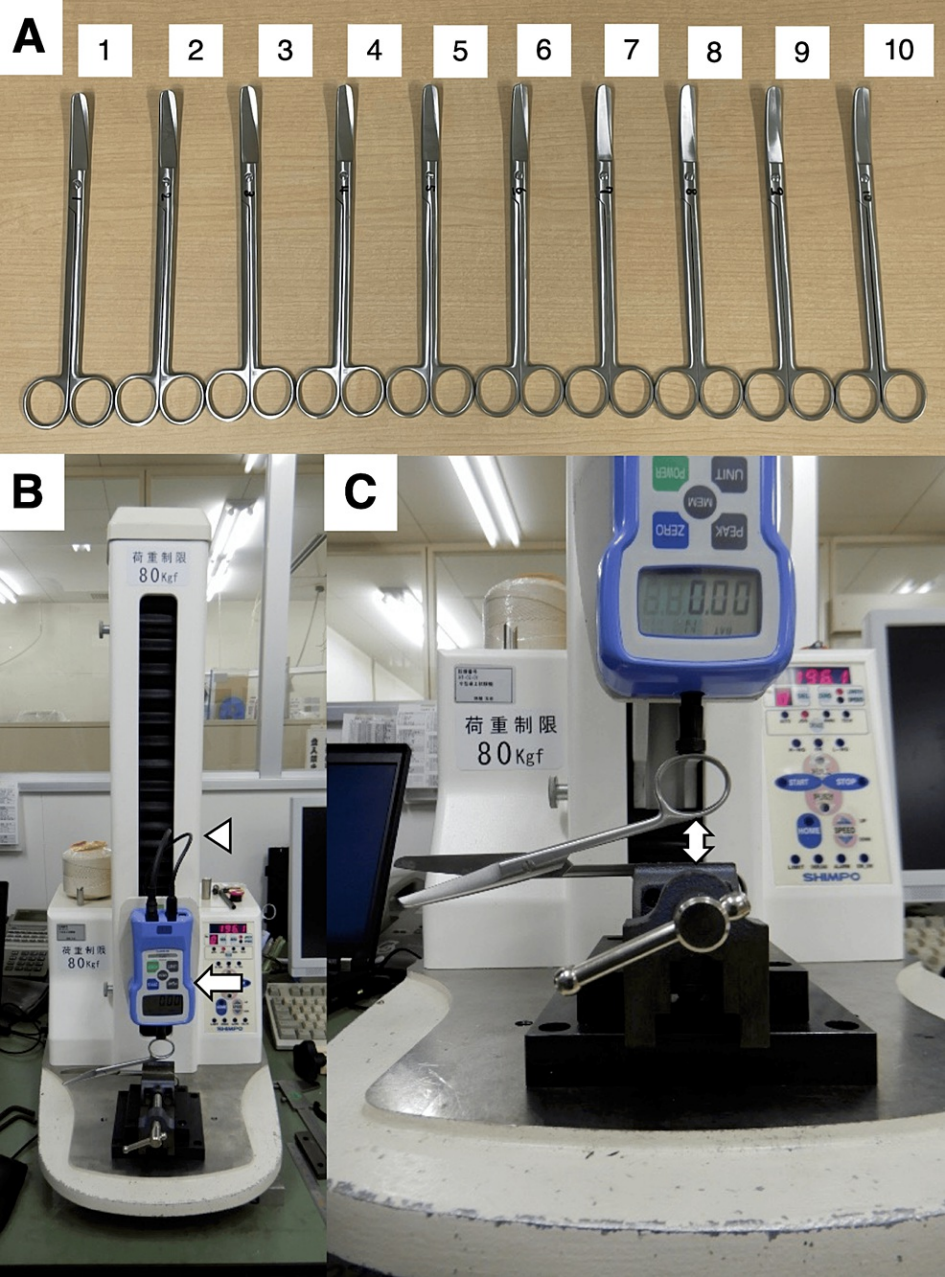
Scissors and measurement test A: Ten pairs of Cooper scissors (20 cm, warped tips, rounded tips, product number: 09-046-00, lot number: TL, stainless steel, MIZUHO Co., Tokyo, Japan) were used in this study. B: The test apparatus included (1) a small tabletop testing machine (arrowhead) and (2) a digital force gauge 20N (arrow). C: The scissors were opened and secured in a vice so that one of the handles was horizontal. The vertical distance from the horizontal position to the top handle position is defined as the "handle width" (double-headed arrow).

Cooper scissors are used frequently and in a variety of ways, such as for dissection, tissue removal, and operative field development [11]. For this reason, they were chosen for this study. Scissors were obtained from SUNMEDIX Co. (Tokyo, Japan). Ten pairs of Cooper scissors (20 cm, warped tips, rounded tips, product number: 09-046-00, lot number: TL, stainless steel, MIZUHO Co., Tokyo, Japan) produced by the same manufacturer and in the same lot were prepared. Each pair of scissors was numbered 1-10 for identification (Figure 1A).

Sensory test

Thirty-one volunteer surgeons, consisting of two females and 29 males, were selected from Jichi Medical University and its affiliated hospitals. The volunteers' surgical experiences were varied; the median number of years of surgical experience was 20 (range: 3-38). Twenty-six surgeons were board-certified surgeons by the Japanese Society of Gastroenterological Surgery or the Japanese Breast Cancer Society (six gastroesophageal surgeons, 12 hepato-pancreato-biliary surgeons, seven colorectal surgeons, one breast surgeon); the others were under training for the board-certified surgeon. Participants were not told that all scissors were in the same lot from the same manufacturer. To avoid sharing results among participants, all participants performed the sensory test independently and at separate times. No time limits were set. The test supervisor acted as an assistant for tasks and answered questions but did not actively participate.

The sensory test was conducted to evaluate the surgeons' feeling when simply opening and closing the scissors. During surgery, surgeons often informally test the comfort of scissors by opening and closing them before use. The present study's sensory test aimed to replicate the performance evaluation of scissors that surgeons usually conduct informally during surgery. Subjective sensations of scissors were quantified by each surgeon's self-report. Although the Visual Analogue Scale (VAS) and Numerical Rating Scale (NRS) are often used to quantify sensory perception [7,12], those self-report scales can be compared for the same person but are not suitable for comparison with others. Therefore, the score is objectified by comparing it to another objective indicator [7,13]. In the present study, since it is difficult to accurately compare slight differences among scissors with a subjective evaluation, we selected three comfortable pairs of scissors and three uncomfortable ones from a total of ten pairs of scissors. In this way, each pair of scissors was rated on a three-point scale (comfortable, neither, uncomfortable). The subjective evaluation of comfort was objectified by comparing it to an objective index of the load required to close the scissors.

Measurement test

A passive test of the opening and closing motion of the scissors was conducted to investigate their physical characteristics, following Mahvash et al. and Pereira et al. in conducting measurements in which scissors are fixed and closed vertically [4,14]. This method eliminates other forces applied to the scissors in other directions. The measurement test was conducted at the factory of MIZUHO Co. (Gosen, Japan). A tabletop testing machine was used to apply a load to the scissors handle and measure the load pressure and displacement of the width between each handle when the scissors were closed (Figure 1B). The test apparatus included a tabletop testing machine (FGS-TV, NIDEC-Shimpo Co., Kyoto, Japan) and a digital force gauge 20N (FGP-2, NIDEC-Shimpo Co., Kyoto, Japan). The scissors were opened and secured in a vise so that one of the handles was horizontal. The initial opening position of the scissors, starting width, was defined as the position where the scissors were closed by their own weight and the handles stopped (Figure 1C). The width of the handle opening at the initial opening position of the scissors was measured. The vertical distance from the horizontal position to the top handle position was defined as the "handle width" (Figure 1C). Once test conditions were set, a load test was conducted using the tabletop testing machine. A load was applied from the head open position until the scissors were closed entirely. Handle closing speed was 10 mm/min. Load values and displacement of the handle opening width were recorded during the test. Only one test was performed on each pair of scissors.

Statistical analysis

The results of the sensory test were scored as 1 point for scissors that felt comfortable, and -1 point for those that felt uncomfortable, and finally, the total number of points received for each pair of scissors was scored as comfortable and uncomfortable. The sum of the scores was tabulated as the overall score. The score for scissors that felt comfortable was defined as a "comfortable score", the score for scissors that felt uncomfortable as an "uncomfortable score", and the overall score as a "total score".

For the measurement test, the load value required to close the scissors (N) and the displacement of the handle width (mm) were recorded. The measured displacement of the handle width was converted to the handle width by defining the closed position as 0 mm. From these, maximum load value, minimum load value, average load value (mean load value), maximum load value minus minimum load value (change in load value), and change in load value at a handle width of 6 mm or less (change in load value at the tip) were obtained for each pair of scissors. 

The correlation between the results of the sensory test and the measurement test results was calculated. A correlation coefficient (r)>0.2 was considered correlated, >0.4 was considered moderately correlated, and >0.7 was considered strongly correlated. A p-value <0.05 was considered significant. Statistical analysis of data was performed by nonparametric tests (Spearman Rank correlation) and multiple regression analysis using R software (The R Foundation for Statistical Computing, Vienna, Austria, version 4.1.0).

## Results

Sensory test

**Figure 2:**
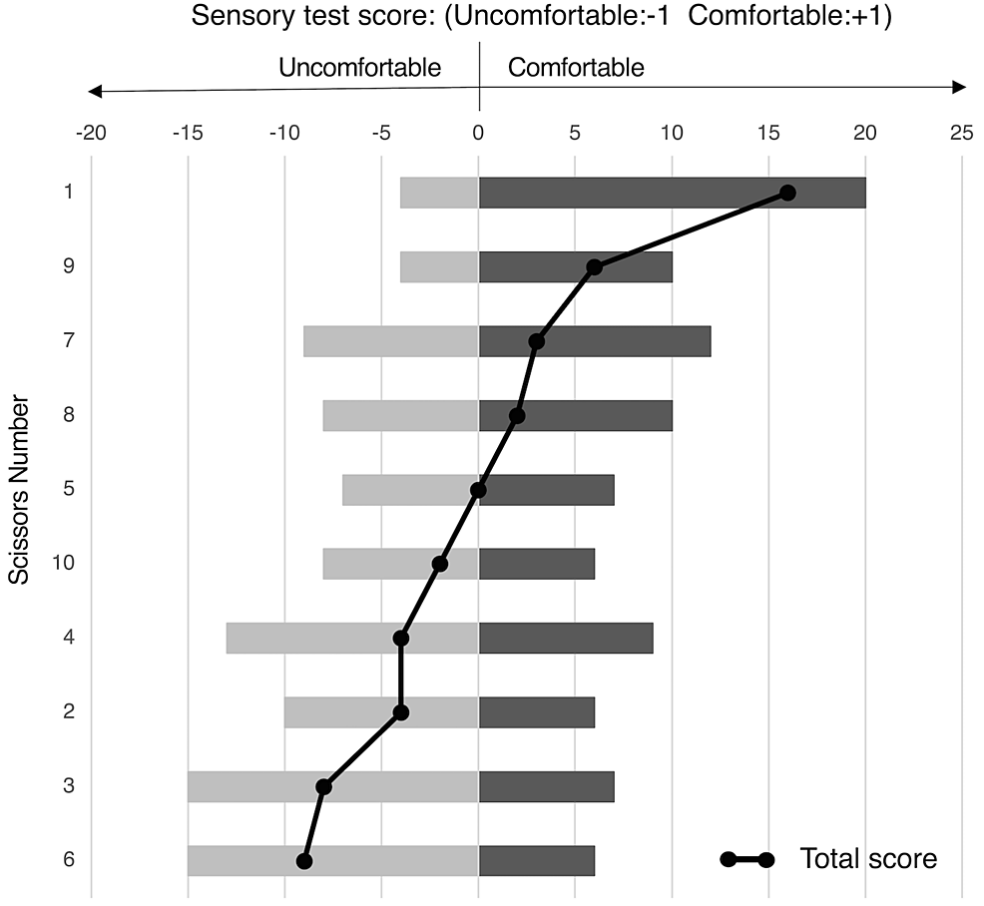
The results of the sensory test Sensory test scores show the number of points scored for comfortable and uncomfortable for each pair of scissors. The scores for comfortable are shown to the right as a dark gray bar, the scores for uncomfortable are shown to the left side in light gray, and the total score by the line graph.

Figure 2 shows the results of the sensory test, in which scissors numbers 1 and 9 had higher total scores, with the most points for comfortable and the fewest for uncomfortable. Scissors 3 and 6 had lower total scores, with the least for comfortable and the most points for uncomfortable.

Measurement tests

Table 1 shows a summary of the measurement test, starting width (mm), maximum load value (N), minimum load value (N), mean load value (N), change in load value (N), change in load value at the tip (N; defined as the difference between the maximum and minimum load values in the range of scissors with a handle opening of 6 mm or less). To facilitate understanding of the relationship to the sensory tests, scissors are listed in the same order as in Figure 2, in descending order of total score on the sensory test.

**Table 1:** Summary of load measurement test results To facilitate correlation with sensory tests, scissors are listed in the same order as in Figure 2, in descending order of total score.

Scissors number	Starting width (mm)	Maximum load value (N)	Minimum load value (N)	Mean load value (N)	Change in load value (N)	Change in load value at the tip (N)
1	20	0.17	-0.11	0.0084	0.28	0.18
9	23	0.30	-0.07	0.0118	0.37	0.35
7	18	0.20	-0.08	0.0291	0.28	0.28
8	18	1.03	-0.02	0.5637	1.05	0.31
5	19	0.30	-0.10	0.0119	0.40	0.40
10	15	0.47	-0.08	0.0308	0.55	0.53
4	15	0.18	-0.09	0.0228	0.27	0.26
2	13	0.36	-0.33	0.0853	0.39	0.38
3	21	1.18	-0.05	0.4316	1.23	0.70
6	17	1.28	0.00	0.6133	1.28	0.62

The median score for each parameter included a starting width of 18 mm (range: 13-23), maximum load value of 0.33 N (range: 0.17-1.28), minimum load value of -0.08 N (range: -0.11-0), mean load value of 0.03 N (range: 0.01-0.61), change in load value of 0.40 N (range: 0.27-1.28), and change in load value at the tip of 0.37 N (range: 0.18-0.70). 

Correlation between the sensory and measurement tests

**Figure 3:**
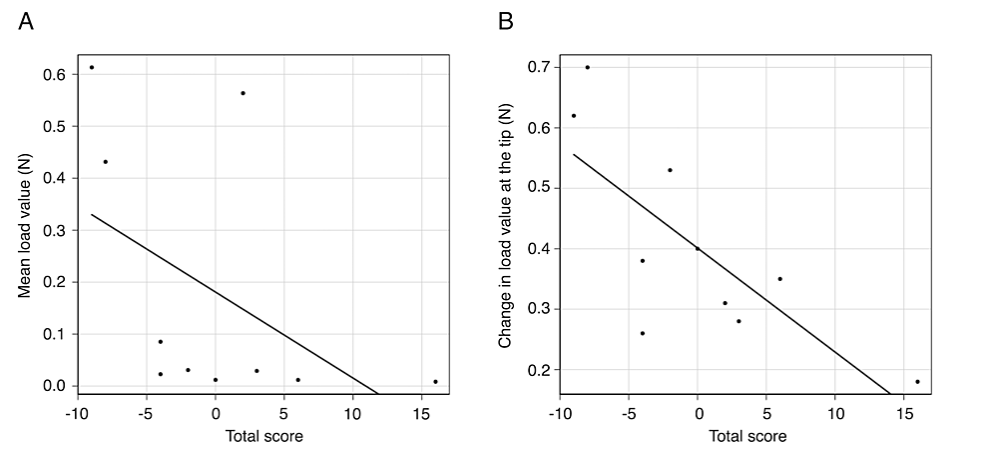
Correlation between the sensory and measurement tests A: Correlation between the total sensory test score and the mean load (r=-0.717, p=0.0195). B: Correlation between the total sensory test score and the change in load at the tip (r=-0.687, p=0.0282).

A strong negative correlation was found between the total score and the mean load in the sensory and measurement tests (r=-0.717, p=0.0195; Figure 3A). The correlation between the total score and the change in load at the tip showed a moderate negative correlation (r=-0.687, p=0.0282; Figure 3B). 

Table 2 shows the results of multiple regression analysis. The change in load at the tip was an independent factor affecting the total score in the sensory test (p=0.046). 

**Table 2:** Results of multiple regression analysis of the total score * p<0.05

Independent variables	Estimate	Standard error	t	p
Mean load value (N)	-2.929	8.55	-0.343	0.742
Amount of change in load value at the tip (N)	-31.201	12.864	-2.425	0.046*

## Discussion

Scissors are one of the most important instruments used in surgery, and improving their utility is essential to improve surgical outcomes. The objective of the present study was to investigate the relationship between surgeons' comfort with scissors and their objective physical properties by conducting a sensory test (to measure comfort subjectively) and a measurement test (to evaluate forces applied to the scissors objectively). Finally, the correlation between scores obtained in the sensory test and the measurement test was investigated. The results showed that the lower the mean load, the more comfortable a surgeon felt when opening and closing the scissors, and the less the change in load values at the tip, the more comfortable the surgeon felt. Multivariate analysis also showed that the change in load at the tip was more influential on comfort than the mean load value.

Although there have been few studies on scissors used in surgery, this has recently gained interest due to the need for VR and robotic surgery instrument development. Greenish et al. [3] and Mahvash et al. [4] have studied and attempted to model the forces applied to scissors during cutting. Greenish et al. mention that tactile differences consist of a combination of large force differences and low-frequency texture components [3]. This study demonstrated that a lower mean load value and a smaller change in load value correlate with increased surgeon comfort. 

The results of the present study show that surgeons sense the force transmitted to the hand from scissors and rate their comfort. In particular, the amount of change in load at the tip is a contributing factor in sensing the quality of scissors. Although the load value was high when using scissors number 8, the change in load at the tip was small, resulting in a favorable evaluation by the surgeon. Waga et al. evaluated the relationship between mechanical stimuli and tactile sensation when cutting with scissors and claimed that sharpness is sensed by slight changes in force when closing scissors and by a sudden decrease in pressure when finishing the cut [7]. Those results support the findings of the present study.

Funahashi et al. reported that vibration and sound feedback in scissors used in VR environments are effective in providing a sense of reality [5]. Okamura et al., in a study that modeled VR scissors with tactile feedback in terms of friction, motion, and physical properties, showed that users could feel the cutting force as if the model were actual scissors, even if the cutting force was not very precise [6]. These results are consistent with the results of the present study that the mean load from the scissors themselves is perceived when using scissors in surgery. 

In recent years, Kansei engineering has been applied to product development and safety improvement in the automotive and electronics industries [15,16]. For hairdressing scissors, Boyles et al. have studied the ergonomic design of scissors handles with the aim of reducing the burden on the user [17]. For fabric scissors, Adeleye et al. and Dianat et al. have similarly studied handle design to reduce the burden on the user [18,19]. Improving the usability of scissors is essential to maximize surgical efficiency and safety. Ergonomic interventions in the surgical environment are known to maximize surgical efficiency and safety [8,9]. Shimomura et al. designed a new handle for scissors used in surgery, evaluated its effectiveness in terms of usability, and reported that this new handle design reduced muscle strain and improved work efficiency [20]. To the best of our knowledge, there have been studies of tactile sensation and ergonomic burden reduction, but no studies to date on comfort during use. Garmer et al. argued that improved user interfaces for medical devices lead to a lower accident rate and reduced instrument learning time [10]. The relationship between comfort and physical characteristics as perceived by surgeons using the scissors is not clear, and we first examined the differences that surgeons feel when opening and closing scissors before actually cutting with the scissors. This is expected to support future quality control standards for the design and manufacture of scissors used in surgery, such as what kind of scissors surgeons prefer and what makes a pair of scissors more usable and comfortable.

The present study investigated the effect of different loads on surgeons' comfort using scissors. To evaluate this, we conducted an experiment using scissors manufactured in the same lot with the same product to eliminate differences in conditions other than load as much the same as possible. It became subjectively obvious that scissors, even from the same lot, had a different feel when opened and closed, likely due to differences during manufacturing. The presence of these differences is not well known. Although each manufacturer inspects their products according to their own standards before shipment, differences in the manufacturer's quality standards and differences perceived by surgeons could be the origin of different feelings. Perhaps surgeons perceive more delicately the differences that the manufacturer considers not to affect product use. The results of the present study may help reduce the effect of these differences, especially if these results are used to assure similar function among scissors produced by a manufacturer.

The present study has acknowledged limitations. This study was conducted with a single type of scissors and by surgeons at a limited number of institutions. In actual surgery, various surgeons use various types of scissors for different purposes. In the future, scissors of different shapes and lengths should be evaluated by surgeons at multiple institutions. In addition, since this evaluation was based on one criterion, "comfortable" or "uncomfortable", it is desirable to conduct a sensitivity evaluation using multiple evaluation items. Further research is needed to examine the physical characteristics of scissors of different shapes and lengths, conduct sensitivity evaluations by a larger number of surgeons, and compare the results to develop standards for scissors used in surgery. 

## Conclusions

Surgeons evaluate Cooper scissors with a low mean load value required to close the scissors and a small change in load value at the tip to be more comfortable. The mean load value, and especially the change in load value at the tip, should be considered in scissor design and manufacturing as a quality standard. Favorable values of these parameters are expected to reduce stress and physical strain on the surgeon during surgery, resulting in increased intraoperative surgeon comfort.
